# African swine fever virus does not express viral microRNAs in experimentally infected pigs

**DOI:** 10.1186/s12917-018-1601-2

**Published:** 2018-09-03

**Authors:** Fernando Núñez-Hernández, Gonzalo Vera, Armand Sánchez, Fernando Rodríguez, José I. Núñez

**Affiliations:** 1 Institut de Recerca i Tecnologia Agroalimentàries (IRTA)-Centre de Recerca en Sanitat Animal (CReSA), Campus de la Universitat Autònoma de Barcelona, Bellaterra, Barcelona, Spain; 2 Centre de Recerca en Agrigenòmica (CRAG), Consorci CSIC-IRTA-UAB-UB, Bellaterra, Barcelona, Spain; 3 Departament de Ciència Animal i dels Aliments, Universitat Autònoma de Barcelona (UAB), Bellaterra, Barcelona, Spain

**Keywords:** African swine fever virus, microRNA, Viral miRNA

## Abstract

**Background:**

African swine fever virus (ASFV) is the etiological agent of African swine fever (ASF), a re-expanding devastating and highly lethal hemorrhagic viral disease. microRNAs (miRNAs) are a new class of small non-coding RNAs that regulate gene expression post-transcriptionally. The discovery of virus specific miRNAs has increased both in number and importance in the past few years. We have recently described the differential expression of several porcine miRNAs during in vivo infection with attenuated and virulent ASFV strains. Here, we have extended these studies trying to identify the presence of viral miRNAs encoded by ASFV in an in vivo infection in pigs.

**Results:**

Sixteen small RNA libraries were analyzed from spleen and submandibular lymph nodes obtained from eight pigs, seven infected with either the virulent E75 ASFV strain or its attenuated counterpart E75CV1, or from pigs surviving E75CV1-infection and challenged with BA71 (heterologous challenge) and one non infected as negative control. Samples were recovered at different times post-infection. Libraries were analyzed by next-generation sequencing. Some viral miRNA candidates were initially identified, which did not correspond to porcine miRNAs. Further structural analyses were carried out in order to confirm if they met the conformational requirements to be considered a viral miRNA.

**Conclusions:**

The analysis of sixteen small RNA libraries prepared from two different tissues obtained from pigs experimentally infected with E75, E75CV1 or with E75CV1 plus BA71, revealed the presence of six potential miRNA sequences but none of them met the requirements to be considered as viral miRNAs. Thus, we can conclude that ASFV does not express miRNAs in vivo, at least under the experimental conditions described here.

## Background

African swine fever (ASF) is a contagious disease that affects domestic and wild pigs caused by ASF virus (ASFV) belonging to the *Asfarviridae* family, genus *Asfivirus*. ASFV is a large icosahedral, enveloped, double stranded (ds) DNA virus ranging from 170 to 193 kb [1]. ASFV is today considered one of the main biological threats for pigs world-wide, causing enormous economical loss in affected countries. Endemic since its origin in several areas of sub-Saharan Africa, ASFV re-entered the European continent in 2007, expanding to surrounding areas and reaching the European Union (EU) borders in 2014. Since then, the virus reached some Eastern European Union countries and in 2017, ASFV was reported in Czech Republic and Romania [2]**.** Lack of vaccines or treatments complicates its control [3], therefore gaining knowledge about ASFV pathogenesis is valuable for the future development of anti-ASFV strategies. The viral particle, with a diameter of 200 nm, has three concentric wraps with an additional external membrane obtained when coming out by budding from the infected cell. Between 150 and 175 ORFs have been identified but the functions for half of them still remain unknown. Sixteen different genomes have been identified and fully sequenced from pigs, wild boars and ticks and 23 genotypes have been so far described [4]. The virus infects monocytes and macrophages, key cell players for the immune system. In addition, it has been observed that virus replicates in endothelial cells, hepatocytes, renal tubular epithelial cells and neutrophils, mainly at late stages of an acute ASFV-infection. miRNAs are a new class of small non-coding RNAs with posttranscriptional regulation functions of gene expression. miRNAs play an increasing role in many biological processes [5]. The study of miRNA-mediated host-pathogen interactions has emerged in the last decade mainly due to the important role that miRNAs play in antiviral defense [6]. In addition, miRNAs encoded by viruses have been identified to be involved in the infection process. To date, the microRNA database, miRBase [7], shows 308 precursors and 502 mature viral miRNAs have been identified. Viruses encoding miRNAs has been identified in several families, as *Herpesviridae, Polyomaviridae, Adenoviridae, Papillomaviridae, Baculoviridae, Ascoviridae* and *Iridoviridae* [8–10]. All of them are DNA viruses, with an essential nuclear phase in their replication cycle, necessary for canonical miRNA biogenesis. In addition, *Ascovirus* and *Iridovirus*, together with *Asfivirus*, belong to the monophyletic superfamily of nucleocytoplasmic large DNA viruses (NCLDV). Thus, ASFV was a good candidate for a virus capable of encoding miRNAs. On the other hand, recently, our group has identified differential expression of porcine miRNAs in pigs after the infection with ASFV [11].

Considering this background, the aim of the present study was to explore if ASFV can encode viral miRNAs in an experimental infection in its natural host by using high-throughput sequencing. To this end, in order to characterize the potential presence of ASFV-specific miRNAs, in silico prediction was carried out to determine if the ASFV genomes of two different ASFV strains with different degrees of virulence, encode possible miRNA precursors. Subsequently, to explore whether the possible miRNAs candidates are present in the ASFV, small RNA libraries from target tissues were constructed and analyzed.

## Methods

### In silico prediction

The Vmir prediction algorithm [12] was used to predict the presence of hairpin structures in the ASFV genomes from: strain E75 (Genbank accession number FN557520) [1] and BA71 (Genbank accession number KP055815) [13]. The sequence of the non-pathogenic BA71V (Genbank accession number NC_001659) [14], highly adapted to Vero cells, was additionally included in the study. Computational prediction was conducted by using Vmir of viral miRNAs, setting as conditions a hairpin size from 50 to 100 nt and a window count of 15.

### Samples analyzed

In order to explore whether these candidates were present in the ASFV genome, samples from spleen and submandibular lymph node (SLN) from seven 8-week-old Landrace X Large White male pigs were used. The animals were acquired from Hypor (A Coruña, Spain), and were fed ad libitum with conventional compound feed for growing pigs. The animals were intramuscularly inoculated in the neck with the virulent ASFV strains: BA71 or E75 or with the attenuated E75CV1, obtained by adaptation of E75 after three passages in Vero cell lineage. The same dose, 10^4^ hemadsorbing units (HAU_50_), was used for each virus. One non-infected pig was included as negative control. All samples used are summarized in Table 1, noticing that some of the samples were previously used to successfully identify the expression of cellular miRNAs during the ASFV infection in vivo [11]. The potential presence of viral miRNAs in ASFV-infected pigs was analyzed by high-throughput sequencing of small RNA libraries prepared from two tissues recovered in three conditions: i) The animals infected with virulent strain E75 necropsied at 3 and 7 dpi, ii) The animals infected with the attenuated strain E75CV1 and necropsied at 3 and 31 dpi, iii) The animals infected with the attenuated strain E75CV1 and re-inoculated at 31 dpi with the virulent strain BA71 and necropsied at 38 dpi. As negative control, small RNA libraries were sequenced from a non-infected animal at 0 dpi.

**Table 1:** Summary of the animals, samples and viral strains used in this study

Animal number	Tissue	ASFV strain/inoculation			Necropsy (dpi)
		E75	E75CV1	BA71^a^	
1	Spleen	+			3
	SLN	+			3
2	Spleen	+			7
	SLN	+			7
3	Spleen		+		3
	SLN		+		3
4	Spleen		+		3
	SLN		+		3
5	Spleen		+		31
	SLN		+		31
6	Spleen		+	+	38
	SLN		+	+	38
7	Spleen		+	+	38
	SLN		+	+	38
8	Spleen				0
	SLN				0

^a^Re-inoculated animals with 10^4^ HAU of the BA71 strain at day 31

The time points chosen for each experimental infection were selected taking into account previous results [15] and covering acute and chronic phases of infection with different ASFV strains. Euthanasia was performed according to European Directive 2010/63/EU, using a pentobarbital overdose of 60–100 mg/kg administered via the anterior vena cava.

All animal experiments were carried out in the BSL3 facilities at CReSA, and all animal managements were performed in accordance with the guidelines of the Good Experimental Practices. All procedures were approved by the Ethical and Animal Welfare Committee of the Universitat Autònoma de Barcelona with the permit number DMAH-5796.

### Real-time PCR

SYBR Green quantitative PCR (qPCR) was used with the following oligonucleotides: pF1 primer (5′ CCTCGGCGAGCGCTTTATCAC 3′) and pR2 (5′ GGAAACTCATTCACCAAATCCTT 3′), designed in a highly conserved region within the p72 ORF [16]. qPCR mix contained 480 nM each primer, 12.5 μl of Power SYBR®Green PCR Master Mix (Thermo Fisher Scientific) and 2.5 μl of template. Nanopure autoclaved water was added to final volume of 25 μL. Amplification were carried out with these conditions: 10 min at 95 °C, 2 min at 50 °C and 40 cycles of 15 s at 95 °C and 1 min at 60 °C. Triplicates of each sample were used for the qPCR.

### Small RNA library construction

Total RNA extractions were carried out in order to construct small RNA libraries from spleen and SLN samples from the animals infected with E75CV1 necropsied at 31 dpi, the E75CV1-infected animals and re-inoculated at 31 dpi with BA71, necropsied at 38 dpi, and from one non-infected animal as described in [17] with some modifications. A total of 8 small RNA libraries were constructed in a two-step ligation procedure with the 3′ and 5′ adaptors from IDT technologies. Briefly, reverse transcription (RT) was carried out with primer mir5 and PCR amplification with primers A5 and B3 (which included multiplex identifiers at the 5′ end) containing sequences complementary to the 3′ and 5′ adaptors used for miRNA library construction. Ion Torrent adapters were ligated to PCR products and libraries were then amplified with Ion Torrent primers. Libraries were sequenced following the manufacturer’s protocol with Ion PGM™ sequencer (Thermo Fisher Scientific, Massachusetts, USA) at DNA sequencing facilities at CRAG (Centre de Recerca en Agrigenòmica, Universitat Autònoma de Barcelona, Spain). On the other hand, cellular miRNAs expression has been previously detected using some of these libraries thus ensuring that they are valid for the present study.

### Sequence processing scheme and analysis

Strict quality parameters of the individual nucleotide sequence reads has been applied and only those with the Phread base quality scores of Q > 30 in all its bases have been used. Primer sequences were removed and only those insert sequences between 15 and 29 nucleotides were used for further analysis. Sequences aligned with *Sus scrofa* genome were analyzed in [11] since they are porcine miRNAs. Local Blast against ASFV genomes was carried out and Mfold [18] was used in order to evaluate the secondary structure and free energy for the potential miRNA candidates. Likewise, candidates were blasted to the output of Vmir hairpin ASFV structures. In addition, candidates were checked with the algorithm developed in miREval 2.0 for detecting novel miRNAs [19].

## Results

**Fig. 1:**
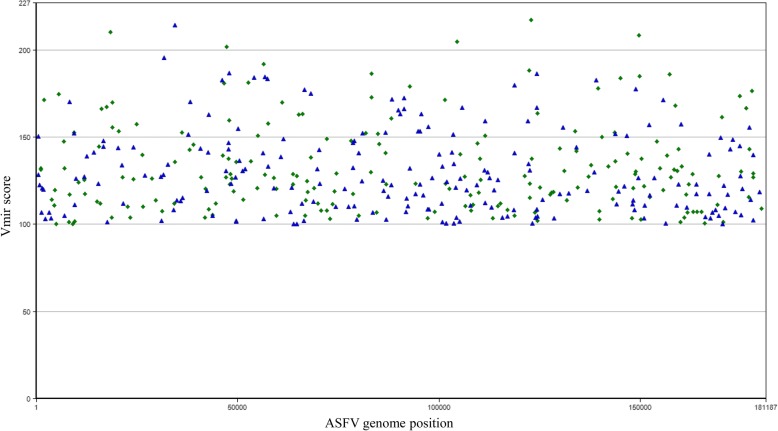
Hairpin structures predicted for ASFV genome of E75 isolate (Genbank accession number FN557520). Predictions have been done by using Vmir with score higher than 100, hairpin size from 50 to 100 and window count of 15. Green diamonds and blue triangles indicate stem-loop structures in direct or reverse orientation, respectively

**Fig. 2:**
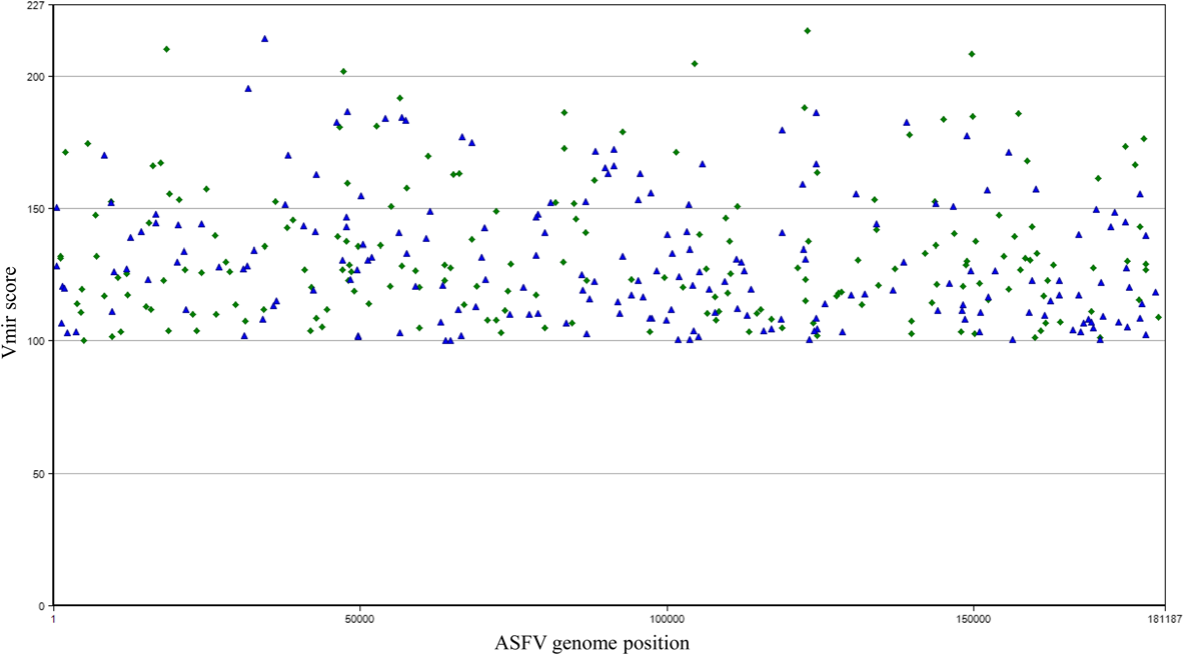
Hairpin structures predicted for ASFV genome of BA71 isolate (Genbank accession number KP055815). Predictions have been done by using Vmir with score higher than 100, hairpin size from 50 to 100 and window count of 15. Green diamonds and blue triangles indicate stem-loop structures in direct or reverse orientation, respectively

The in silico prediction allowed the identification of 415 miRNA candidates in the E75 strain with a score higher than 100 (Fig. 1), in the case of BA71, 391 candidates with a score higher than 100 were identified (Fig. 2) and in the case of BA71V, 366 candidates were identified in the same conditions (data not shown).

In the animal infection, the pigs inoculated with the virulent strains E75 and BA71 developed typical clinical signs, while the pigs inoculated with the attenuated E75CV1 strain developed mild or no clinical signs [15]. Quantitative PCR was performed from the samples of spleen and SLN from all the animals. Those animals inoculated with E75 showed low Ct values at 7 dpi coinciding with the peak of virus replication and ASF clinical signs, while in contrast, inoculation of the same dose of the homologous attenuated E75CV1 strain showed no amplification. The animals infected with E75CV1 and re-inoculated with the BA71 virulent strain showed amplification levels similar to those found after E75 infection, correlating with the lack of cross-protection observed for these two strains [15]. As expected, the negative control animal did not show any specific PCR amplification (Table 2).

**Table 2:** qPCR from spleen and SLN of the eight animals in the in vivo infection

Tissue	Animal number							
	1	2	3	4	5	6	7	8
Spleen	31.99	27.81	–	–	–	24.84	27.3	–
SLN	27.71	24.6	–	–	–	25.17	28.11	–

Ct values from animals inoculated with the E75 virulent strain at 3 and 7 dpi (animals 1 and 2); animals inoculated with the E75CV1 attenuated strain and necropsied at 7 and 31 dpi (animals 3, 4 and 5); animals re-inoculated at 31 dpi and necropsied at 38 dpi (animals 6 and 7) and negative control animal sacrificed at 0 dpi (animal 8)

Total RNAs from SLN and spleen were used to construct small RNA libraries from the selected animals. High-throughput sequencing provided 272,967 reads from the eight new libraries that, in addition to eight previously sequenced libraries with 301,802 reads, provided a total of 574,769 reads from the sixteen libraries (Table 3). New sequencing data were submitted to ENA database [20] with the accession numbers ERX1811688–95. When trimmed to filter sequences ranging from 15 to 29 nucleotides, a total of 169,511 reads were obtained. After the identification of host miRNAs by comparing with miRBase [7], sequences were blasted to ASFV. Reads were blasted to the E75 and BA71 isolates considering only sequences with 100% of alignment and identity rendering 9 hits with the ASFV genome (Table 3). An increased number of mismatches in the extremes due to miRNA variability (isomiRs) [21] were allowed. The search rendered 31 hits. This processing allowed the identification of 40 viral genome hits with 8 unique sequences when allowing 100% homology (Tables 3 and 4) and 23 unique sequences when allowing < 100% homology (Tables 3 and 5). From these sequences, 25 candidates were obtained when grouped by isomiRs and 6 candidates when copy number (CN) was ≥2.

**Table 3:** Summary of the high-throughput reads, blast and candidates for viral miRNAs discovery

Total reads	574,769
Trimmed not- empty reads (15–29 nt)	169,511
Blast ASFV 100%	9
Unique sequences 100%	8
Blast ASFV < 100%	31
Unique sequences < 100%	23
Total unique sequences	31
Viral miRNA candidates^a^	25
Viral miRNA candidates CN ≥ 2	6
Candidate + Mfold structure/ ASFV miRNAs	–

^a^ Viral candidates copy number (CN) after sequence association by isomiRs

**Table 4:** Candidates obtained with a 100% of identity with the ASFV genome and their copy number (CN)

Candidate ID	Candidate sequences blast vs ASFV = 100%	Candidates CN
ASFV-C1	GAGACCAAGAACCTGGGCA	2
ASFV-C2	ATGTGCCCTGGTCCCGTAGGAGTG	1
ASFV-C3	CGTCTGGGAGAAATGGAGCA	1
ASFV-C4	CACCGTGTGGATGCCCAGGG	1
ASFV-C5	GAGCTCGTGACCATTGAA	1
ASFV-C6	GGTTCACTGGTGTCCATGAT	1
ASFV-C7	ATGATCGTAGCATTTGGTGTA	1
ASFV-C8	GGCGAGTCACTTGGTTTGCT	1

**Table 5:** Candidates obtained with less than 100% of identity with the ASFV genome and their copy number (CN) grouped by isomiRs

Candidate ID	Candidate sequences blast vs ASFV < 100%	IsomiRs CN	Candidates CN
ASFV-C9	TCTCCCAACCTGTACAGT	4	9
	TCTCCCAACCTGTACAGGT	1	
	CTCCCAACCTGTACAGT	3	
	TCGTCCCAACCTGTACAGT	1	
ASFV-C10	TGAGGTAGTAGGTTGTGT	2	4
	TGAGGTAGTAGGTTGTGTG	2	
ASFV-C11	CGCTGCGGGTGTGGTGGGCC	1	2
	CGCTGCGGGTGTGGTGG	1	
ASFV-C12	TGTGCAAATCTATGCAAAAC	2	2
ASFV-C13	GGTAGTAGGTTGTGTGGTT	1	2
	GGTAGTAGGTTGTGTGGAT	1	
ASFV-C14	AGGGCACCAAGGTAAC	1	1
ASFV-C15	ACGCCCTGGGGCCTATGAG	1	1
ASFV-C16	TCGCTGCGGGTGTGGTGGG	1	1
ASFV-C17	CCGCTGCGGGATGAAC	1	1
ASFV-C18	GGGAAAATCCACGGCCCTGC	1	1
ASFV-C19	TGTATGGAGGCCCTTTTT	1	1
ASFV-C20	CTCCTGGGGCCGCACTCTC	1	1
ASFV-C21	TTCACTGCCCTGATCCTCT	1	1
ASFV-C22	GCGACCCGTCAAGTCCAACAG	1	1
ASFV-C23	GTCGGAGCCTGAGCCGGGAG	1	1
ASFV-C24	CCCCCACACAGTTTGAC	1	1
ASFV-C25	TCTCCCAATCCTTGCCAGT	1	1

Sequences were blasted to the output of Vmir hairpins from E75, BA71 and BA71V. In addition, candidates were evaluated with miREval 2.0 software with no positive results. Therefore, after the analysis of the complementarity of the candidates with the Vmir prediction and the checking with miREval, together with the analysis with Mfold of their secondary structure and the free minimum energy of their folding, none of the candidates could be considered as a viral miRNA as they failed to fulfill the necessary requirements.

## Discussion

In a previous study carried out by our group, it was described that there is in vivo upregulation and downregulation of several porcine miRNAs after infection with ASFV [11]. Using the small RNA libraries obtained for this purpose plus new small RNA libraries constructed in this work, we have studied the capability of E75, BA71 and E75CV1 isolates of ASFV to express viral miRNAs in vivo by using high-throughput sequencing.

The genomic characteristics of ASFV fit with the requirements of a virus to be able to encode viral miRNAs because it is a DNA virus with a nuclear phase [22, 23] and the in silico prediction carried out in this work found several potential hairpins to be virus- encoded miRNAs. In addition, 16 viral miRNAs have been identified in Singapore grouper iridovirus (SGIV) [24] and in tiger frog virus (TFV) [25] both members of the *Iridoviridae* family. Likewise, virus encoded miRNAs have been identified in ascovirus which infects mosquitoes [26]. In this last case, a viral miRNA reduces DNA polymerase I levels by transcriptional degradation and modulates the ascovirus replication. All these viruses belong, as the asfivirus, to the monophyletic superfamily of nucleocytoplasmic large DNA viruses (NCLDV), reinforcing that ASFV is a good candidate to explore virus encoded miRNAs.

There is no clear knowledge about when viral miRNAs are expressed at highest levels after infection according to Cullen [27], who showed the variability of the viral miRNAs expression with time among different viruses. Therefore, we have analyzed samples from the infected animals at different times points post-infection in addition to different viruses with different virulence and the animals infected with an attenuated strain and re-inoculated with a heterologous virulent strain in order to increase the spectrum of conditions. Besides, although E75 and BA71 are classified in the West African-European cluster, the percentage of identity at genome level is 88.5%, the lower identity among viruses included in this cluster [1]. ASFV produces lytic infection. Nevertheless, although most virus-encoded miRNAs have been related to persistent infections, viral miRNAs have also been detected during lytic infection [9, 17].

After the identification of porcine miRNAs expressed in ASFV-infected animals [11], high-throughput sequencing revealed other potential miRNAs sharing identity with the viral genome. Out of a total of 40, six with a copy number ≥ 2 were selected for further analysis. These candidates were blasted against the Vmir hairpins prediction from E75 and BA71 genomes. Also, BA71V, a highly adapted strain in Vero cells, was included in the analysis in order to avoid the loss of candidates because of the possibility of genome variability in cell culture passages [13]. However, it was observed that none of the candidates matched any of the predicted hairpins, had appropriate secondary structure, or minimum free energy, showing that the candidates did not have the necessary characteristics to be identified as viral miRNAs. The candidate ASFV-C9, which presents the higher CN (9), mapped into the early structural protein p22 [28]. p22 is a target gene of miR-30e-5p, a differentially expressed (DE) miRNA described in our previous work [11]. This would explain why a higher number of sequences have been found. On the other hand, all isomiRs were blasted to miRBase, and none of them were identified as host miRNA. Therefore, ASFV-C9 with all other sequences can be considered as degradation products of viral RNA. Being aware of the reduced number of animals used, different tissues were used to generate a total of 14 RNA libraries, number that exceeds the majority of similar studies so far published, normally ranging from 1 to 4 and carried out in cell culture, and not in the natural host [29–32].

Although DNA viruses have the potential to encode viral miRNAs, this characteristic does not imply the viral miRNAs expression of these viruses. For example, the herpesvirus family have the highest viral miRNAs encoding rates, and almost each member is capable to codify miRNAs. However, there are members of this family as the varicella zoster virus (VZV) and other human neurotropic herpesviruses (HSV1 and HSV2) [33], which do not encode viral miRNAs. The knowledge of why some viruses encode miRNAs and why some phylogenetically related viruses do not, can be of great relevance for the fields of virology and RNAi [10].

In this study, we have found that ASFV, under these experimental conditions, does not express viral miRNAs like other viruses such as Cowpox virus or Porcine circovirus type 2 (PCV2) [34, 35]. Interestingly, in a recent and exhaustive work carried out by Jaing et al. about gene expression in blood of ASFV-infected pigs, wherein some DE host miRNA have been identified, viral miRNAs are not described [36]. In the literature, there are examples in which were indicated that certain viruses encode miRNAs that were initially considered as non coding miRNAs, e.g. human papillomavirus [37]. Therefore, studies with higher number of reads, together with analysis of the presence of viral miRNAs in cell culture and other in vivo conditions should be considered before fully discounting if ASFV expresses miRNAs.

## Conclusions

In the present work, we can conclude that African swine fever virus does not express viral miRNAs in the in vivo conditions studied here. This finding is contrary to what happens with other large DNA viruses and can be relevant to the biology of this important pathogen.

## Abbreviations

*ASF*: African swine fever
*ASFV*: African swine fever virus
*CN*: Copy number
*DE*: Differentially expressed
*DPI*: Days post-infection
*ENA*: European nucleotide database
*HTS*: High-throughput sequencing
*NCLDV*: Nucleocytoplasmic large DNA virus
*OIE*: World Organization for Animal Health
*SLN*: Submandibular lymph node
